# Genome-Wide Gene by Environment Interaction Analysis Identifies Common SNPs at 17q21.2 that Are Associated with Increased Body Mass Index Only among Asthmatics

**DOI:** 10.1371/journal.pone.0144114

**Published:** 2015-12-16

**Authors:** Leyao Wang, William Murk, Andrew T. DeWan

**Affiliations:** Department of Chronic Disease Epidemiology, Yale School of Public Health, New Haven, Connecticut, United States of America; Cincinnati Children's Hospital Medical center, UNITED STATES

## Abstract

Asthmatics have an increased risk of being overweight/obese. Although the underlying mechanisms of this are unclear, genetic factors are believed to play an essential role. To identify common genetic variants that are associated with asthma-related BMI increase, we performed a genome-wide gene by environment (asthma) interaction analysis for the outcome of BMI in the Multi-Ethnic Study of Atherosclerosis (MESA) study (N = 2474 Caucasians, 257 asthmatics), and replicated findings in the Framingham Heart Study (FHS) offspring cohort (N = 1408 Caucasians, 382 asthmatics). The replicable tagging SNP, rs2107212, was further examined in stratified analyses. Seven SNPs clustered in 17q21.2 were identified to be associated with higher BMI among asthmatics (interaction p < 5×10^−7^ in MESA and p < 0.05 in FHS). In both MESA and FHS asthmatics, subjects carrying the A allele on rs2107212 had significantly higher odds of obesity than non-carriers, which was not the case for non-asthmatics. We further examined BMI change subsequent to asthma diagnosis over a period of 26 years in FHS and demonstrated greater BMI increase among asthmatics compared to non-asthmatics. Asthmatics carrying the A allele at rs2107212 had significantly greater net BMI increase over the 26-year period compared to non-asthmatics. In this study, we found that common genetic variants on 17q21.2 are associated with post-asthma BMI increase among Caucasians. This finding will help elucidate pathways involved in the comorbidity of asthma and obesity.

## Introduction

Asthma and obesity are two rapidly growing public health issues, and the comorbidity of these conditions poses an enormous burden on asthma control as well as quality of life [1]. The simultaneously increasing prevalence of both asthma and obesity suggests a potential intrinsic link between these two chronic disorders [2, 3], and a recent longitudinal study identified asthma as a risk factor for subsequent obesity [4]. However, the underlying factors that contribute to this relationship have remained largely unknown.

Genetic factors play an essential role in both asthma and obesity and are believed to be predominantly responsible for the comorbidity of the two conditions [5–7]. A genomic inversion in 16p11.2 was identified to be protective against the joint occurrence of asthma and obesity in adults of European decent [8]. This provided genetic evidence of asthma-obesity co-occurrence. In terms of single nucleotide polymorphisms (SNPs), a candidate gene association study was conducted to identify shared genetic variants between childhood asthma and obesity, but no SNP was associated with both phenotypes among previously identified asthma and BMI genes [9]. This implied that SNPs underlying the comorbidity of asthma and obesity may exist in novel loci. A genome-wide analysis study (GWAS) was also conducted to identify genetic variants associated with BMI among 23,000 asthmatics [10]. A SNP in *DENND1B* was identified in asthmatic children in the discovery dataset but was not replicable. These two SNP studies primarily targeted childhood asthma. A potential explanation of the non-significant findings may be that the association between BMI and asthma is not as strong as in adults, since no significant BMI difference between asthmatics and non-asthmatics was observed at baseline in the GWAS study.

To further explore the genetic influences of asthma-related obesity, we aimed to identify genetic factors that lead to elevated BMI in an asthma-dependent manner. A gene by environment interaction model could increase the power to detect SNPs associated with BMI not discovered when modification by asthma is ignored. Therefore, we performed a genome-wide SNP by asthma interaction analysis for BMI on subjects from the Multi-Ethnic Study of Atherosclerosis (MESA) data set, and subsequently attempted to replicate genome-wide suggestive interacting regions in an independent dataset, the Framingham Heart Study (FHS) offspring cohort. We then evaluated the most significant interacting SNP for association with greater BMI and obesity risk only among asthmatics. To examine the causal direction of the asthma-BMI association, we further examined BMI change over a 26-year period after asthma diagnosis in FHS and demonstrated the risk from asthma to subsequent obesity and a susceptibility locus that links them.

## Materials and Methods

### Subjects and phenotype definition

The Multi-Ethnic Study of Atherosclerosis (MESA) and its ancillary study, MESA Air, were used for analysis [11]. MESA is a population-based study focusing on characteristics and risk factors of subclinical cardiovascular disease (CVD). The study is comprised of 6,814 men and women aged 45–84 who were free of clinical cardiovascular diseases, recruited through six field centers across the United States. The screening exam (exam1) took place in 2000 and was followed by four examinations (exams 2–5) in 2002, 2004, 2005 and 2010 respectively. Data from exams 1–4 were available at the start of the project and were used for analysis. Height and weight were measured at every visit and the BMI value (BMI = weight (kg)/(height (m))^2^) from the screening exam was used in the present analysis. Asthma-related questions for exams 1–4 are listed in (Table A in S1 File). Asthmatics were defined as those who reported doctor-diagnosed asthma in the first exam; non-asthmatics were defined as those who never reported doctor-diagnosed asthma or asthma medication use in all four exams. We included all participants in the first examination who identified themselves as Caucasian (n = 2527). Subjects with missing information on asthma phenotype (n = 10), inconsistent phenotype information (i.e. those who used asthma medication but did not report doctor-diagnosed asthma, n = 134), or incident asthma after first examination (i.e. those who first reported asthma in exams 2–4, n = 34) were excluded. MESA Air added 253 new participants, and after exclusion of subjects with missing information, we included 249 additional subjects. Of the remaining subjects from both MESA and MESA air (n = 2598), 2588 were genotyped as part of the MESA SNP Health Association Resource (MESA SHARe) and were included for further analysis (Fig A in S1 File).

Data from the Framingham Heart Study (FHS) was used for replication of the top regions from the discovery stage [12]. The objective of FHS is to identify common factors or characteristics that contribute to CVD. It was established in 1948 and has developed as a prospective, community-based, three-generation study. We selected participants in the offspring cohort for analysis since this population had a similar age distribution as the MESA population. There were a total of eight exams from 1971 to 2005, and height and weight information was collected at each visit. BMI, calculated based on the height and weight information in the eighth examination (BMI = weight (lbs)/height (in)^2^ ×703), was used in the present analysis. Except for the first exam, self-reported asthma and clinical diagnostic impression of asthma (CDI asthma) questions were asked at each visit and are listed in (Table B in S1 File). Asthmatics were defined as those who ever had self-reported asthma or wheezing in any exams. Non-asthmatics were defined as those who never had self-reported asthma and wheezing or CDI asthma. We included subjects with non-missing height and weight information in the eighth examination (n = 2852). Subjects with CDI asthma but not self-reported asthma (n = 13), without genotype data (n = 178), or not confirmed as Caucasians (n = 311) were removed, resulting a total of 2350 subjects included in further analysis (Fig A in S1 File).

### Ethical statement

Data for both MESA and FHS were obtained from the database of Genotypes and Phenotypes (dbGaP) (MESA accession number: phs000209.v12.p3; FHS accession number: phs000007.v23.p8). This study was approved by Yale University Human Investigation Committee. Patient records/information were anonymized and de-identified prior to analysis.

### Genotyping and quality control

In MESA, genomic DNA was isolated from peripheral blood samples and genotyped on the Affymetrix Genome-Wide Human SNP Array 6.0 chip containing 909,622 SNPs. Quality control procedures and subsequent genetic analyses were performed using PLINK 1.07 [13]. Nineteen subjects were removed because their overall call rate was <98% and six subjects were removed because the reported sex did not match the genotyped sex as called in PLINK. SNPs were excluded if they met any one of the following criteria: 1) not mapped to an autosomal chromosome (n = 37,380); 2) call rate <98% (n = 21,669); 3) the genotype distributions of the SNP deviated from those expected by Hardy–Weinberg equilibrium (HWE) at p < 1×10^−7^ (n = 1,729); 4) monomorphic or minor allele frequency (MAF) <1% (n = 126,951). A subset of linkage disequilibrium (LD)-pruned SNPs was generated using pairwise r^2^>0.5 in a window of 50 SNPs and shifted by 5 SNPs at each step for genetic quality control procedures, including identity-by-decent (IBD) and principal components analyses. The pairwise IBD matrix was calculated to assess cryptic relatedness. Thirty-three pairs of subjects were identified that had a pi-hat value > 0.2. One subject from each pair was randomly removed. Principal component analysis was conducted using default parameters in EIGENSTRAT 3.0 [14] to address population stratification. Fifty-six outliers were identified after five iterations and excluded from further analysis. A total of 2474 subjects (n = 257 asthmatics, n = 2217 non-asthmatics) with 721,893 SNPs were included in the genome wide interaction association analysis (discovery stage).

In the Framingham SNP Health Association Resource (SHARe), a sub-study of FHS, subjects were genotyped on the Affymetrix 500K mapping array plus Affymetrix 50K supplemental array, with 500,568 SNPs in total. The same quality control procedures and criteria as used in MESA were applied to the FHS dataset. Subjects with call rate <98% (n = 277) and subjects with unmatched sex check (n = 8) were removed. SNPs that were not mapped to an autosomal chromosome (n = 12,422), had a call rate <98% (n = 79,984), failed HWE (n = 1,776), or had MAF <1% or were monomorphic (n = 57,689) were excluded. LD-pruned SNPs from the remaining SNPs were used for genetic quality control. One subject from each pair with pi-hat value >0.2 was randomly removed (n = 563), and outliers from the principal component analysis (n = 94) were removed. A total of 1408 subjects (n = 382 asthmatics, n = 1026 non-asthmatics) and 348,697 SNPs were included in the replication stage analysis.

### Genetic analysis

For genetic analysis, SNPs were coded as an additive genetic model (0, 1, and 2, indicating the number of minor alleles), and BMI was treated as a quantitative trait. Asthma status was dichotomously coded. Population stratification was inspected through the genomic inflation factor (λ) in single SNP analyses, with adjustment for age, sex, asthma status and increasing numbers of principal components. We found that the first principal component was sufficient to account for population stratification. Quantile-quantile (QQ) plots based on p-values from the genome-wide single SNP analyses are shown in (Fig B in S1 File). A genome-wide SNP by asthma interaction analysis was then conducted on MESA subjects, with a linear model that included main effect terms for the SNP and asthma, and an interaction term for SNP × asthma. The model also included covariates to adjust for age, sex, and the first principal component. Strict Bonferroni-corrected significance was defined as p < 6.93×10^−8^ (0.05/721,893), while genome-wide suggestive significance was defined as p<5×10^−7^, for tests of interaction terms. Three genetic regions that each had more than three genome-wide suggestive SNPs in high LD (r^2^ > 0.8) were identified, and the SNP in each region with the smallest number of missing subjects was selected as a tag SNP for replication in FHS.

In FHS, a genome-wide single SNP analysis was first performed with a linear regression model adjusted for age, sex, asthma status and the first principal component to inspect genomic inflation (Fig B in S1 File). For replication of interactions involving the tag SNPs in FHS, SNP-asthma interactions for BMI as the outcome were analyzed using linear regression models as already described. Interactions reaching nominal significance (p<0.05) in FHS were considered potentially replicated. The regional plot for the replicable region, 17q21.2, was made using LocusZoom [15]. The rest of the six genome-wide suggestive SNPs in 17q21.2 were then tested for interaction in FHS. SNP imputation was performed using IMPUTE2 [16] with default settings for the two un-genotyped SNPs (rs12601191 and rs16968877) in FHS. HapMap 3 haplotypes with NCBI build 36 (hg18) coordinates were used as the reference panel. The imputation had an overall concordance rate of 96.9% and the info score was 0.988 for rs12601191 and 0.979 for rs16968877. All SNPs were then examined and subjected to meta-analysis, conducted using the R package “meta” [17], with both fixed effect and random effect models. The most significant interacting SNP from the meta-analysis, rs2107212, was selected for the stratification analysis.

### Stratified analysis

The association between genotypes of rs2107212 and BMI was evaluated in asthmatics and non-asthmatics in MESA and FHS subjects. A qualitative analysis using obesity (defined as BMI> = 30 kg/m^2^) vs. non-obesity (BMI < 30 kg/m^2^) as the outcome was performed. A logistic regression model with adjustment for age, sex and the first principal component was used to evaluate the odds ratio and 95% confidence interval (CI) of being obese for each additional risk allele, stratified by asthma status, in MESA and FHS and in a pooled meta-analysis. Since there was no significant heterogeneity between these studies (Cochrane’s Q test p>0.05 and I^2^<10%), a fixed effect model was used in the meta-analysis. We also evaluated the association when using different BMI cut-offs of 28 kg/m^2^ and 25 kg/m^2^ to define obesity. In FHS, a subset of subjects who participated in exam 2 and had BMI and asthma information were extracted to examine their BMI change from 1979 (exam 2) to 2005 (exam 8). Genotype-specific mean BMI at both time points were calculated. Net BMI change over 26 years was then compared between genotypes with or without the risk allele, stratified by asthma status. Welch’s t-tests were applied when comparing the difference of two groups.

## Results

### Summary of participants

Summary statistics of participants from MESA (n = 2474) and FHS (n = 1408) following quality control are shown (Table 1). The distribution of sex, age and BMI were similar for the two data sets. Asthma prevalence differed between the two studies (10% vs. 27% in MESA and FHS, respectively). BMI was significantly higher in asthmatics compared to non-asthmatics in both studies (Welch’s t-tests). As expected, the prevalence of obesity was significantly higher among asthmatics compared to non-asthmatics in both data sets (1 degree of freedom chi-square test).

**Table 1:** Characteristics of subjects in MESA and FHS. Participants who were included in the final interaction analyses are summarized. In MESA, age is based on the screening exam; in FHS, age is based on the 8^th^ exam with 5-year intervals. Classification was based on BMI, as follows: underweight (BMI<18.5); normal weight (18.5< = BMI<25); overweight (25< = BMI<30); obesity (BMI> = 30).

Variables	MESA (n = 2474)			FHS (n = 1408)			
	asthma	non-asthma	p-value		asthma	non-asthma	p-value
	(n = 257)	(n = 2217)			(n = 382)	(n = 1026)	
Age (Mean ± SD)	62.91±10.12	61.04±9.65			68.08±8.82	68.77±8.83	
Male (%)	115 (45%)	1097 (49%)			189 (49%)	476 (46%)	
BMI (Mean ± SD)	29.01±6.04	27.59±6.04	3.25×10^−4^		29.17±5.72	27.74±4.90	1.75×10^−5^
Classification							
Underweight	3	12			4	10	
Normal weight	66	716			82	298	
Overweight	89	910			153	431	
Obesity (%)	99 (39%)	579 (26%)	3.37×10^−5^		143 (37%)	287 (28%)	7.73×10^−4^

### Genome-wide SNP by asthma interaction analysis

**Fig 1:**
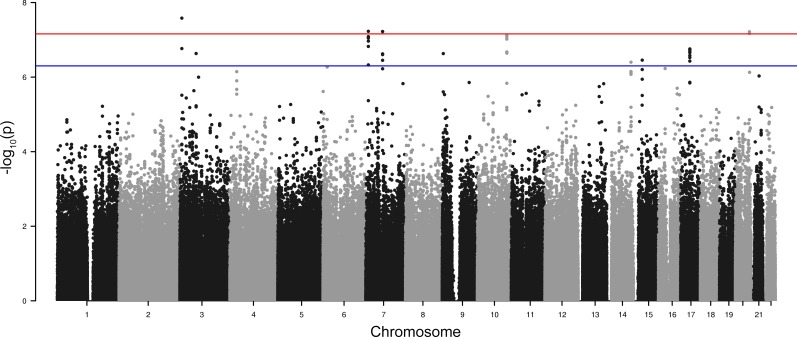
Manhattan plot of interaction p-values derived from the MESA genome-wide interaction analysis. Linear regression models, including main effects for SNP and asthma, and an interaction term for SNP × asthma, were used. Models were also adjusted for age, sex, and the first principal component. The red (upper) line represents the genome-wide significant p-value (0.05/721,893 = 6.93×10^−8^). The blue (lower) line represents the genome-wide suggestive p-value of 5×10^−7^.

**Fig 2:**
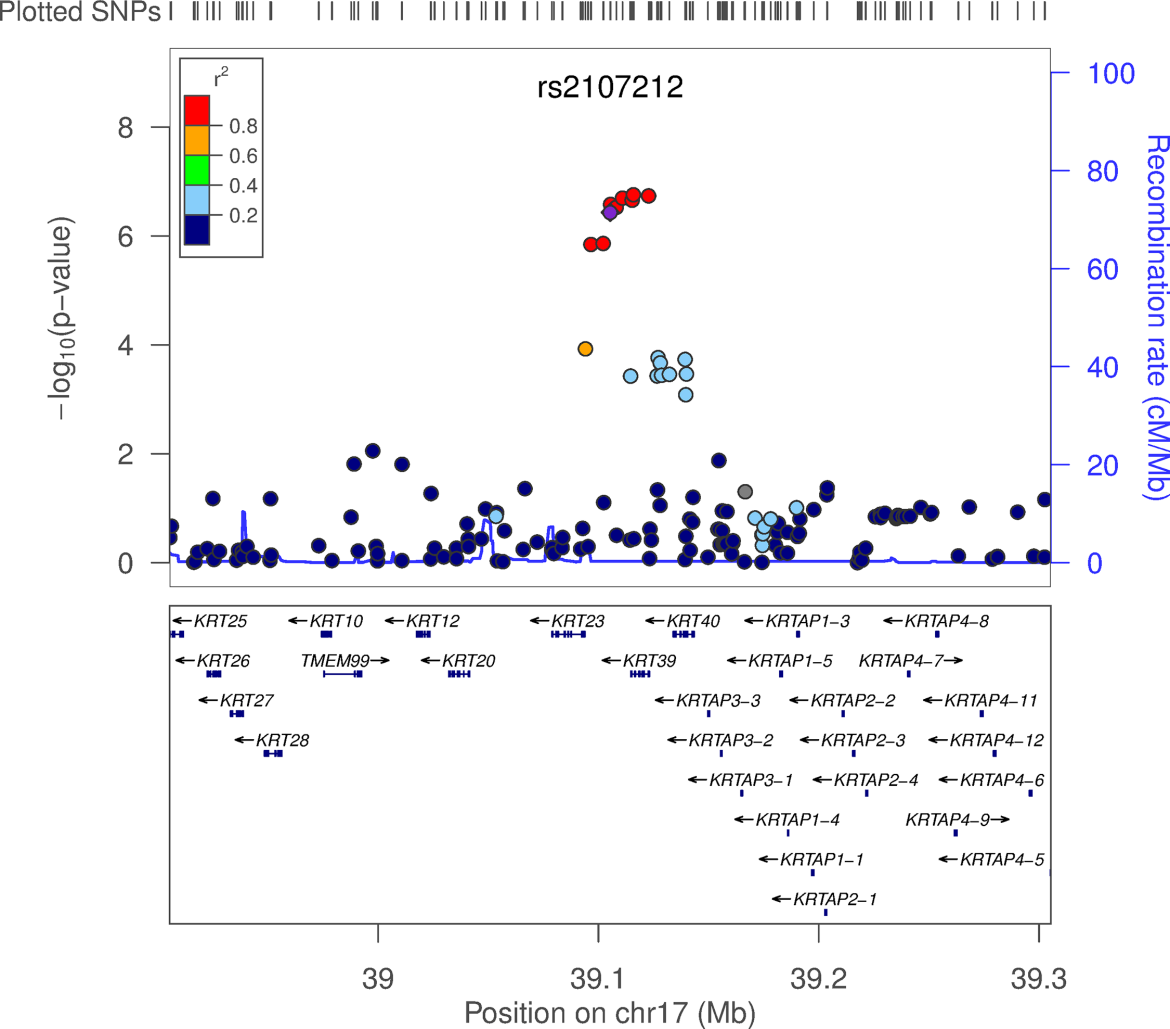
Regional plot showing replicable SNPs in 17q21.2. A regional plot was generated in LocusZoom Version1.1 for the MESA genome-wide interaction analysis results. P-values on the -log10 scale are displayed on the y axis, and chromosome positions are displayed on the x axis. This plot shows the tag SNP (rs2107212) in purple with a 200kb flanking region on each side. The pairwise LD pattern with rs2107212 is also shown. The bottom panel shows the name, position and transcription direction of each gene in this region.

Genome-wide SNP by asthma interaction analysis was first performed on MESA subjects. The linear regression model included main effects for SNP and asthma, and an interaction term for SNP × asthma. The model was also adjusted for age, sex, and the first principal component. Overall interaction results are shown in the Manhattan plot (Fig 1). Thirty-one interacting SNPs exceeded the genome-wide suggestive threshold of p < 5×10^−7^ (Table C in S1 File), and three genetic regions that each contained more than three genome-wide suggestive interacting SNPs in high linkage disequilibrium (LD) with each other were selected: 7p21.3, 10q25.3 and 17q21.2. The SNPs with the least missing subjects in each region were chosen as tag SNPs for further validation in FHS. Of the three tag SNPs, one SNP (rs2107212 in 17q21.2) had a significant SNP by interaction p-value in replication subjects (p = 9.38×10^−3^), while the other two SNPs did not reach the replication significance threshold of 0.05 (Table 2). To further verify the significant region, we then tested all seven MESA genome-wide suggestive SNPs in 17q21.2 in FHS. We observed that all seven SNPs had a SNP by asthma interaction replication p-value < 0.05, in the same direction as observed in MESA. The p-values for the seven SNPs ranged from 1.76×10^−7^ to 3.72×10^−7^ in MESA and 9.38×10^−3^ to 0.047 in FHS (Table D in S1 File). The regional plot for SNPs located within 200kb of the tag SNP, rs2107212, in MESA is presented (Fig 2), where a clear cluster of SNPs in strong LD is shown. SNP rs2107212 was also the most significant asthma-interacting SNP in this region following a meta-analysis of the MESA and FHS data (p = 2.51×10^−3^ under a random effect model and p = 5.60×10^−8^ under a fixed effect model) (Table D in S1 File).

**Table 2:** Top interacting regions associated with BMI and replication in FHS. From the MESA gene by environment interaction analysis, regions with more than three genome-wide suggestive SNPs (interacting p-value <5×10^−7^) in strong LD (r^2^ >0.8) were selected as top interacting regions. SNPs with fewer missing subjects in each region were chosen as tag SNPs to replicate in FHS. SNP position was based on the GRCh38 assembly.

SNP	Region	Position	Gene/Location	Dataset	β (95%CI)	p-value
rs10250689	7p21.3	10120482	*HSPA8P8/330kb up*	MESA	4.66 (2.98, 6.34)	5.91E-08
				FHS	0.56 (-0.98, 2.10)	4.75E-01
rs7904383	10q25.3	115716396	*ATRNL1/Intron*	MESA	-2.66 (-3.63, -1.70)	7.42E-08
				FHS	-0.06 (-0.94, 0.81)	8.87E-01
rs2107212	17q21.2	40949109	*KRT39/9kb down*	MESA	2.96 (1.82, 4.09)	3.72E-07
				FHS	1.49 (0.37, 2.61)	9.38E-03

### Association between BMI and rs2107212 genotypes

**Fig 3:**
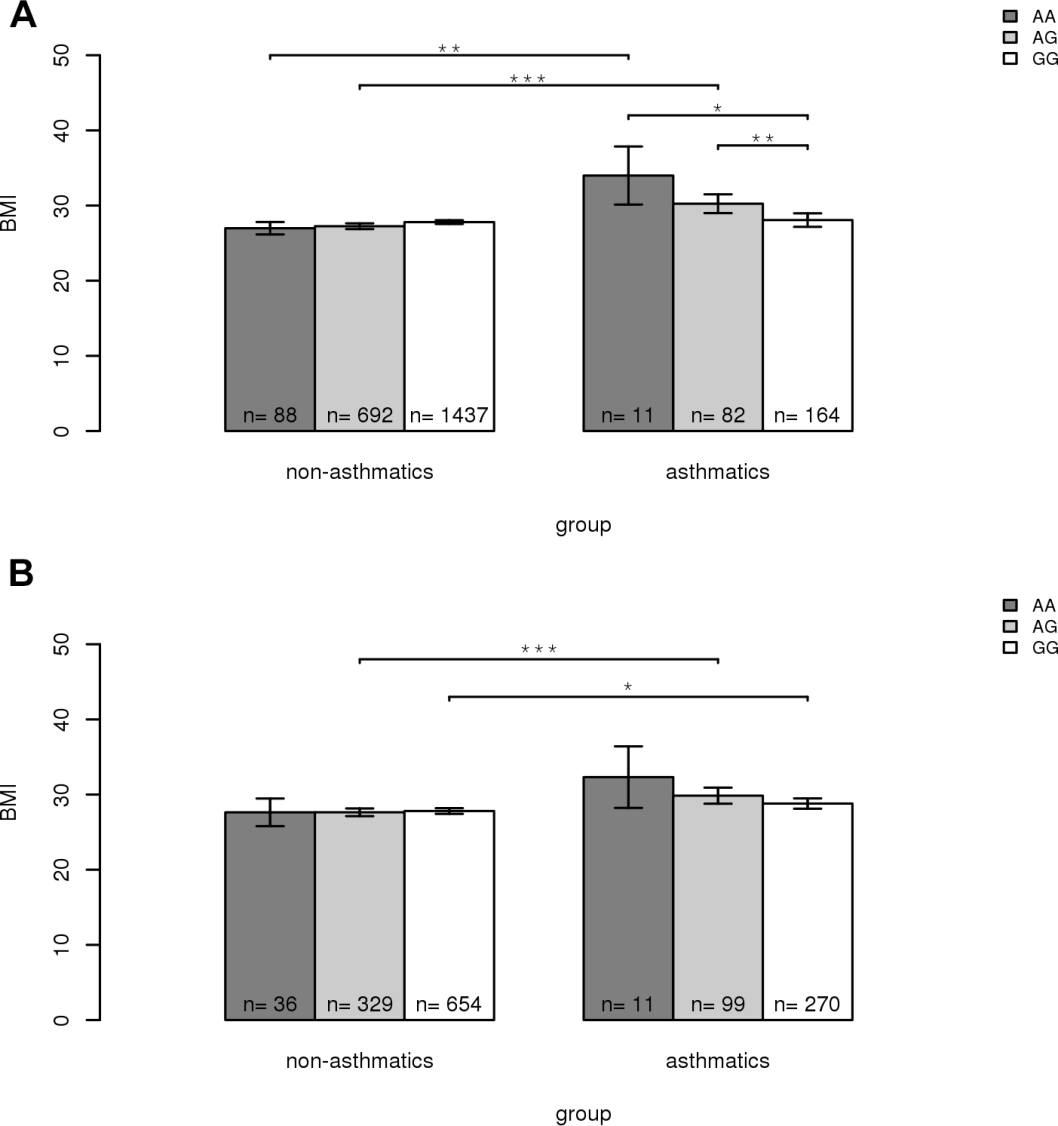
Association of rs2107212 genotypes and BMI, stratified by asthma status in MESA (A) and FHS (B), respectively. Body mass index (BMI, kg/m^2^) was compared across subjects carrying the AA, AG or GG genotypes for rs2107212 in asthmatics and non-asthmatics. Numbers of subjects are shown in each bar. Data are represented as the mean ± SEM. Significance was tested using Welch’s t-test (*p<0.05, **p<0.01 and ***p<0.001).

To further explore the nature of the interaction, we conducted a series of stratified analyses for rs2107212 among the asthmatic and non-asthmatic populations in MESA and FHS. First, we evaluated the association between rs2107212 and BMI stratified by asthma status in MESA (Fig 3A and Table E in S1 File). Among asthmatics, increasing copies of the A allele at rs2107212 were associated with successively higher BMI values. Interestingly, among non-asthmatics, an opposite trend was observed, although the differences were not significant. Subjects carrying the AA or AG genotype had significantly higher BMI among asthmatics compared to non-asthmatics. In FHS, we observed a similar trend for the effect of the A allele (Fig 3B and Table E in S1 File). Subjects carrying the AG genotype had a significantly greater BMI difference between asthmatics and non-asthmatics, compared to those carrying the GG genotype. Subjects carrying the AA genotype had higher BMI among asthmatics compared to non-asthmatics, although the p-value did not reach nominal significance (p = 0.06).

### Evaluation of the odds ratio of being obese for the A allele at rs2107212

**Fig 4:**
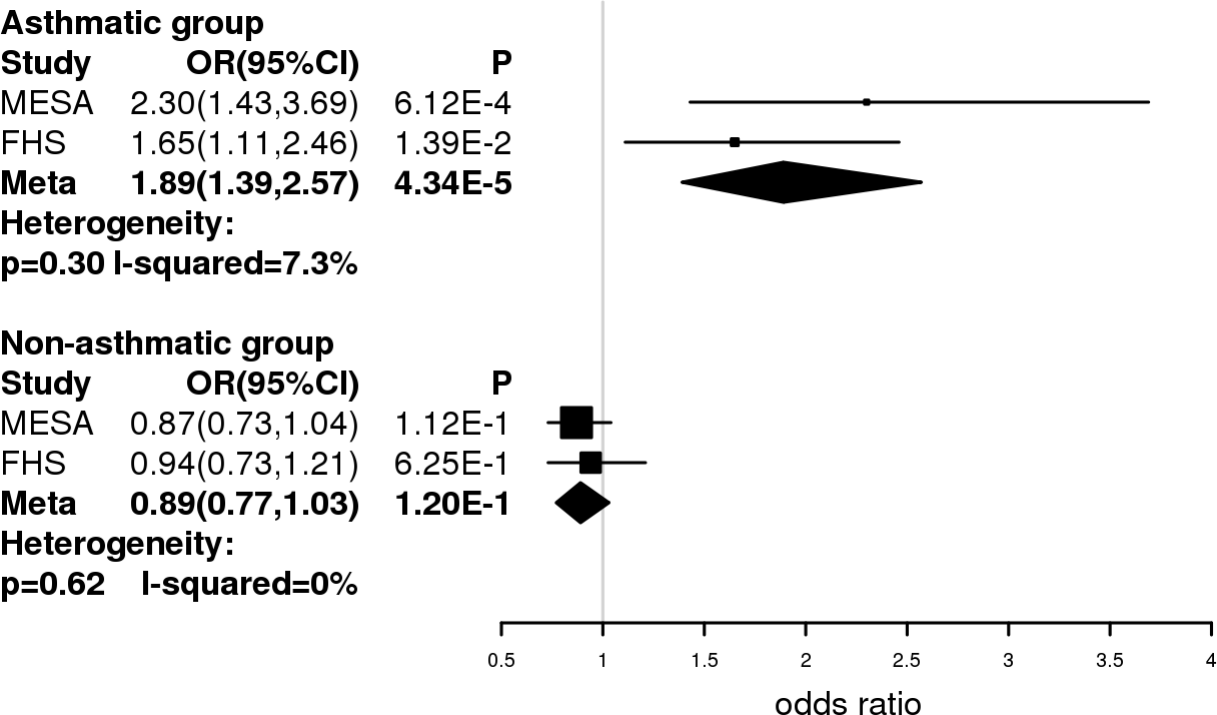
Forest plot showing the association of rs2107212 with obesity in MESA, FHS and meta-analysis. Asthma status-specific odds ratios (OR) of being obese for rs2107212 were calculated using logistic regression models adjusted for age, gender and the first principal component in MESA and FHS respectively. The minor allele A is the “risk” allele, and an additive genetic model was used. Meta-analysis was performed under a fixed effect model since the heterogeneity tests were not significant. Boxes indicate group-specific OR point estimates, and lines indicate the respective 95% confidence interval (CI). Diamonds indicate meta-analysis OR and 95% CI.

We next examined and compared the odds ratio (OR) of being obese (BMI> = 30 kg/m^2^) among the asthmatic and non-asthmatic populations for rs2107212, in MESA and FHS separately as well as together in a meta-analysis. The forest plot showed a consistent effect of the A allele in MESA and FHS (Fig 4). The meta-analysis showed that the overall odds of being obese increased by 1.89 fold for each additional A allele in the asthmatic population. In the non-asthmatic population, the odds of being obese decreased by 0.89 fold per additional A allele; however, this was not significantly different from 1.0. To further test if the asthma-specific association results for the A allele at rs2107212 are still present with varying cut-offs for classifying obesity, we evaluated the association using different BMI cut-offs of 28 kg/m^2^ and 25 kg/m^2^. The results are shown in (Table F in S1 File), from which it is apparent that different BMI cut-offs resulted in similar trends of association for rs2107212. This further demonstrated that the A allele at rs2107212 was associated with BMI increase and obesity incidence only among asthmatics.

### BMI change over a 26-year period after asthma diagnosis in FHS

**Fig 5:**
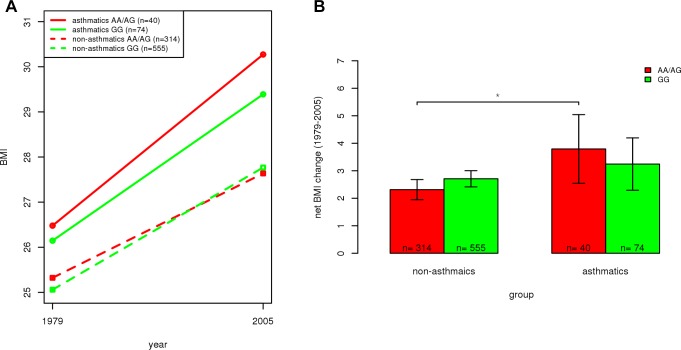
Association of rs2107212 genotypes and BMI change by asthma status in FHS. A: For a subset of FHS subjects who had BMI and asthma information at both exam 2 (1979) and exam 8 (2005) (n = 983), the means of BMI at both time points are shown according to the rs2107212 genotypes AA/AG and GG, stratified by asthma status. B: Net BMI change (BMI value in 2005—BMI value in 1979) with rs2107212 genotypes AA/AG and GG stratified by asthma status is shown. The numbers of subjects are shown in each bar. Data are represented as the mean ± SEM. *p<0.05 under Welch’s t-test.

In FHS, 990 subjects had BMI and asthma data for both exams 2 (1979) and 8 (2005), of which 983 subjects had genotype information for rs2107212. Mean BMI at these two times, stratified by asthma status and rs2107212 genotype, is shown (Fig 5A and Table G in S1 File). BMI among asthmatics was significantly higher than in non-asthmatics in 1979 (p = 0.0102), and the difference was more significant in 2005 (p = 6.84×10^−4^). During this 26-year period, in asthmatics, subjects carrying the AA or AG genotype increased their BMI more than subjects carrying the GG genotype; however, this was not true among non-asthmatics (Fig 5B). During this time period, asthmatics carrying the AA or AG genotypes had a mean BMI change of 3.79, while subjects carrying the GG genotype had a mean BMI change of 3.24. Among non-asthmatics, subjects carrying the AA or AG genotypes had a mean BMI change of 2.31, while subjects carrying the GG genotype had a mean BMI change of 2.71 (Table H in S1 File). This result further indicates that the asthmatic population in general gained more weight than the non-asthmatic population, and that the A allele at rs2107212 increased the BMI change by 60% among asthmatic compared to non-asthmatics (p = 0.0308).

## Discussion

Many correlated disorders have shared genetic backgrounds, and the genetic effects on one of the phenotypes might be modified by the other phenotype. This provides a novel model supporting the integration of gene by environment interaction terms (i.e., where the ‘environment’ is another disease or phenotype) in a GWAS framework to explore comorbid susceptibility genes. These genetic factors could be overlooked when only SNP main effects are examined.

The comorbidity of asthma and obesity is a growing medical problem. While previous studies have focused on the unidirectional relationship of obesity preceding asthma onset, post-asthma BMI increase is a neglected but potentially important phenomenon, particularly since asthma is an early onset disease. Here, we used two independent data sets of older adults and hypothesized that asthma impacted BMI through genetic factors. Therefore, we conducted a SNP by asthma interaction analysis to identify SNPs that are associated with BMI and modified by asthma status. SNPs in 17q21.2 were verified to be associated with higher BMI and higher risk of obesity only when asthma is present.

We further took advantage of the longitudinal FHS data set to obtain BMI measured closest to asthma onset and evaluated BMI change after asthma diagnosis, from 1979 to 2005. (Fig 5A) reflects the trend of BMI change over this 26-year period. In 1979, the asthmatic population already had significantly higher BMI than non-asthmatics. The BMI differential further increased in the intervening 26 years and resulted in a much greater BMI differential among asthmatics than non-asthmatics. When genotype was taken in to consideration, asthmatic subjects carrying the minor allele of rs2107212 gained more weight than subjects not carrying this allele, while this was not the case among non-asthmatics. Since BMI in 1979 was likely measured after but closer to asthma onset than BMI in 2005, we can infer that the BMI difference between asthmatics and non-asthmatics before asthma onset was less than in 1979 and that actual BMI increase after asthma may be greater. In (Fig 5B), we further examined the net BMI change over a 26-year period, where rs2107212 functioned as a risk locus for BMI increase only when asthma was present. Here, we identified a genetic locus that is associated with post-asthma BMI increase and demonstrated that asthma could influence weight gain over a long period.

Chromosome 17q21 has been repeatedly reported to be associated with asthma [18–20]. Although the region covering the ORMDL3, GSDMA and GSDMB genes is the most replicated one, the keratin (KRT) cluster has also been shown to be associated with asthma in a previous GWAS study [21]. KRT proteins are key structural components in epithelial cells and are essential for tissue function. A study identified *KRT18* as a bronchial epithelial autoantigen that is associated with adulthood nonallergic asthma [22]. *KRT18* and some other keratin genes have also been identified to be associated with obesity-caused fatty liver [23, 24]. Hair follicles have been demonstrated to be associated with Toluene diisocyanate (TDI) -induced asthma [25]. Non-functional mutations in filaggrin, a protein binding to and condensing the keratin cytoskeleton, are risk factors for asthma, atopic eczema and allergies [26]. The identified SNP, rs2107212, is located in the intergenic region flanked by keratin genes *KRT39* and *KRT23* (9 kb upstream of *KRT39* and 11 kb downstream of *KRT23*). *KRT39* is a type I keratin gene with expression late in the differentiation of hair [27]. *KRT23* is a type I epithelial keratin gene that has been identified to interact with 14-3-3 proteins to modulate key cellular processes in a *SMAD4*-dependent manner [28]. In addition, *KRT23* has been reported to be associated with several cancers, such as colon cancer, pancreatic cancer and hepatocellular carcinoma [29–31]. This study links the keratin family genes with asthma and obesity phenotypes. However, the underlying biological mechanisms of rs2107212 in asthma-dependent BMI increase needs to be further explored.

This study is potentially confounded by reverse causality from obesity to asthma. Longitudinal studies have observed an increased odds ratio or relative risk of incident asthma among the obese population [32, 33]. We cannot exclude the possibility of a causal effect of obesity on asthma since BMI before asthma development was not available in MESA and the Framingham offspring study. However, to attempt to further assess SNP effects on BMI increase after asthma diagnosis, we used a subset of subjects from the Framingham offspring cohort who had both asthma status and BMI values in 1979 and 2005. Over a period of 26 years, the net BMI change was significantly higher in asthmatics than non-asthmatics. This result demonstrates that SNP rs2107212 is involved in the effect of asthma on BMI. However, from the data we have, we cannot determine if rs2107212 is also involved in the reverse relationship, BMI on asthma.

Another limitation in this study is that MESA and FHS did not use the same asthma definitions. MESA asked about doctor-diagnosed asthma, while FHS asked about wheezing and asthma in the same question and a doctor diagnosis was not required. This could explain the asthma prevalence difference (10% in MESA and 27% in FHS). A relatively looser asthma definition in FHS may have potentially diluted any signals and may have underestimated some of the interactions, which we believed was the primary reason for lack of replication of other top interacting regions. However, the consistency in direction of effect between MESA and FHS for the tag SNPs strongly argues in favor of these being true positive interactions.

After adjusting for the number of SNPs tested in the genome-wide screen, this study had over 80% power to detect an interaction coefficient (β) of 5 when the SNP MAF is ≥ 0.1, and an interaction coefficient (β) of 4 when the SNP MAF is ≥ 0.2 (Table I in S1 File). While this is a modest level of power, this dataset allowed us to identify a novel and replicable gene × environment interaction.

In conclusion, this is the first attempt at a genome-wide interaction analysis to detect genetic risk factors associated with BMI modified by asthma. SNPs in 17q21.2 were identified as risk loci for BMI increase only among asthmatics. This finding will help elucidate pathways involved in the comorbidity of asthma and obesity. Additional studies are needed to further verify and emphasize the impact of asthma on obesity/BMI and examine the underlying mechanisms.

## Supporting Information

S1 File Flow diagram showing subject exclusion and inclusion procedures in MESA and FHS (Fig A). Quantile-quantile (QQ) plots based on p-values from the genome-wide single SNP analyses in MESA and FHS (Fig B). Asthma questions in MESA (Table A). Asthma questions in FHS (Table B). Top interacting SNPs associated with BMI (p-value <5×10–7) from the SNP by asthma interaction analysis in MESA (Table C). Replication results of genome-wide suggestive interacting SNPs at 17q21.2 in FHS and their meta-analysis results from MESA and FHS (Table D). BMI value of rs2107212 genotypes by asthma status in MESA and FHS (Table E). The association of each additional minor allele in rs2107212 and obesity with different BMI cut-offs (Table F). BMI value of rs2107212 genotypes by asthma status in 1979 and 2005 in FHS (Table G). Net BMI change from 1979 to 2005 for rs2107212 genotypes by asthma status in FHS (Table H). Power analysis for MESA (Table I).
(DOCX)
