# Supplementary material for: Genome-Wide Gene by Environment Interaction Analysis Identifies Common SNPs at 17q21.2 that Are Associated with Increased Body Mass Index Only among Asthmatics
Source: PLoS One. 2015 Dec 16;10(12):e0144114. doi: 10.1371/journal.pone.0144114 (PMC4684413; doi:10.1371/journal.pone.0144114)
Supplement: S1 File — Quantile-quantile (QQ) plots based on p-values from the genome-wide single SNP analyses in MESA and FHS (Fig B). Asthma questions in MESA (Table A). Asthma questions in FHS (Table B). Top interacting SNPs associated with BMI (p-value <5×10^–7^) from the SNP by asthma interaction analysis in MESA (Table C). Replication results of genome-wide suggestive interacting SNPs at 17q21.2 in FHS and their meta-analysis results from MESA and FHS (Table D). BMI value of rs2107212 genotypes by asthma status in MESA and FHS (Table E). The association of each additional minor allele in rs2107212 and obesity with different BMI cut-offs (Table F). BMI value of rs2107212 genotypes by asthma status in 1979 and 2005 in FHS (Table G). Net BMI change from 1979 to 2005 for rs2107212 genotypes by asthma status in FHS (Table H). Power analysis for MESA (Table I). (DOCX) [file pone.0144114.s001.docx]

**S1 File supporting information**


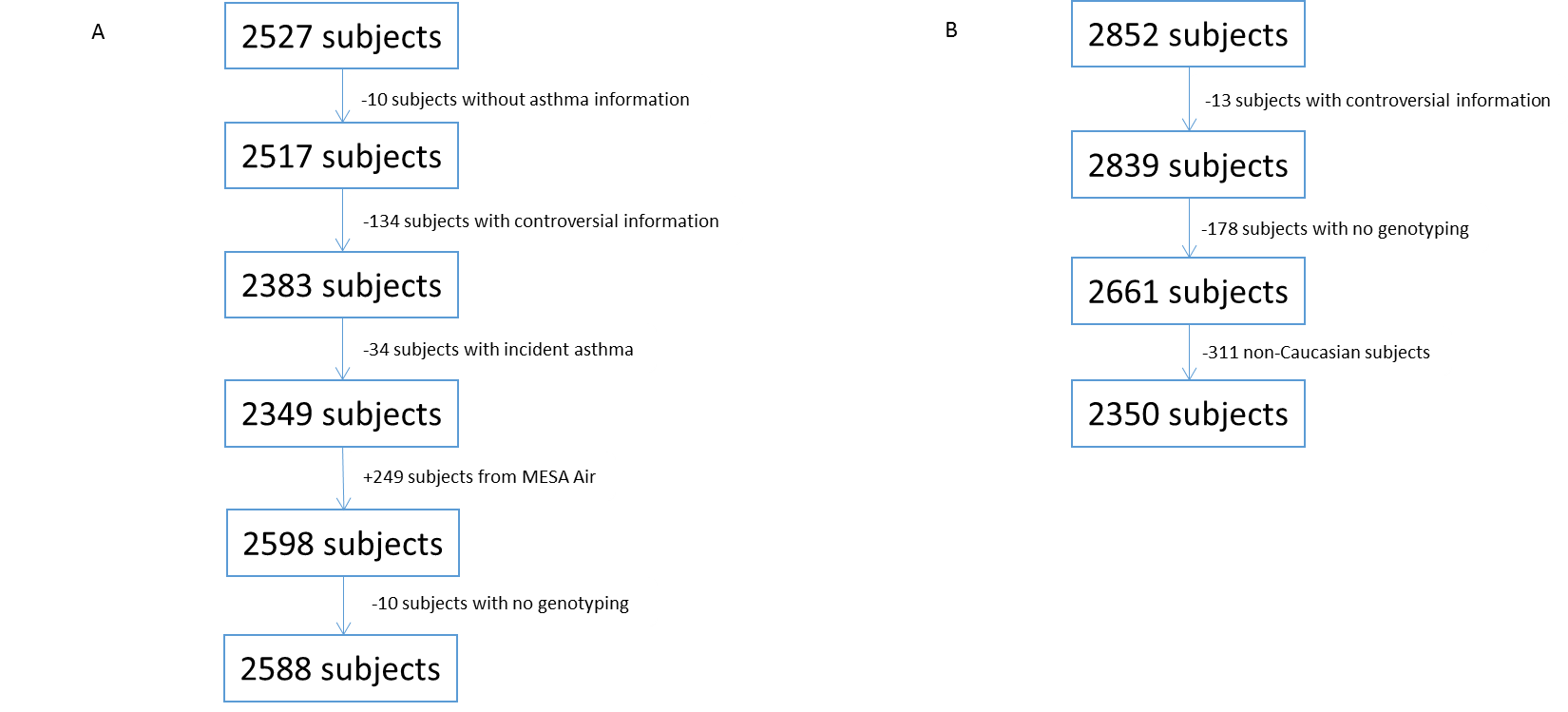
**Fig A. Flow diagram showing subject exclusion and inclusion procedures in MESA (A) and FHS (B)**

**Fig B. Quantile-quantile (QQ) plots based on p-values from the genome-wide single SNP analyses in MESA (A) and FHS (B)**

**
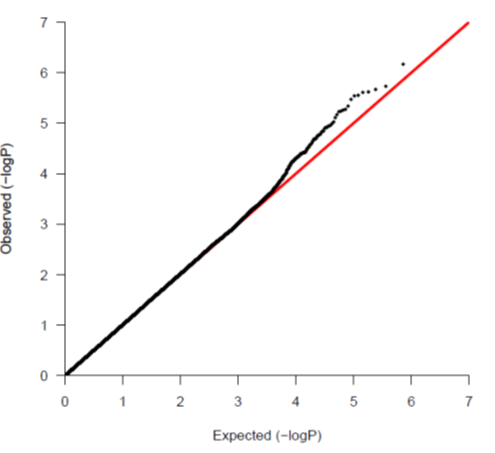
A B**

A linear regression model adjusted for age, sex, asthma status and the first principal component was used in MESA and FHS, respectively. The genomic inflation factor (λ) was **1.0083** in MESA and **1** in FHS.

**Table A. Asthma questions in MESA**

| Exam | Time |  | Questions |
| --- | --- | --- | --- |
| 1 | 2000-2002 |  | Has a doctor ever told you that you had asthma |
|  |  |  | Current medication: Oral anti-inflammatory asthma drugs leukotriene receptor antagonists and inhibitors of lipo-oxygenase |
|  |  |  | Current medication: Inhaled steroids for asthma |
| 2 | 2002-2004 |  | Has a doctor told you that you have developed asthma since your last MESA visit |
|  |  |  | Current medication: Oral anti-inflammatory asthma drugs leukotriene receptor antagonists and inhibitors of lipo-oxygenase |
|  |  |  | Current medication: Inhaled steroids for asthma |
| 3 | 2004-2005 |  | Has a doctor told you that you have developed asthma since your last MESA visit |
|  |  |  | Current medication: Oral anti-inflammatory asthma drugs leukotriene receptor antagonists and inhibitors of lipo-oxygenase |
|  |  |  | Current medication: Inhaled steroids for asthma |
| 4 | 2005-2007 |  | Has a doctor told you that you have developed asthma since your last MESA visit |
|  |  |  | Current medication: Oral anti-inflammatory asthma drugs leukotriene receptor antagonists and inhibitors of lipo-oxygenase |
|  |  |  | Current medication: Inhaled steroids for asthma |
| MESA Air | 2005 |  | Has a doctor ever told you that you had asthma |
|  |  |  | Current medication: Oral anti-inflammatory asthma drugs leukotriene receptor antagonists and inhibitors of lipo-oxygenase |
|  |  |  | Current medication: Inhaled steroids for asthma |

**Table B. Asthma questions in FHS**

| Exam | Time | Questions |
| --- | --- | --- |
| 2 | 1979-1984 | Troubled with wheezing—asthma |
| 3 | 1984-1987 | Wheezing or asthma |
|  |  | Clinical Diagnostic Impression of asthma |
| 4 | 1987-1991 | Wheezing or asthma |
|  |  | Clinical Diagnostic Impression of asthma |
| 5 | 1991-1995 | Wheezing or asthma |
|  |  | Clinical Diagnostic Impression of asthma |
| 6 | 1995-1998 | Wheezing or asthma |
|  |  | Clinical Diagnostic Impression of asthma |
| 7 | 1998-2001 | Have you had asthma in the interim |
|  |  | Have you ever had asthma |
|  |  | Clinical Diagnostic Impression of asthma |
| 8 | 2005-2008 | Since your last exam have you had asthma |
|  |  | Clinical Diagnostic Impression of asthma |

**Table C. Top interacting SNPs associated with body mass index (BMI) (p-value <5×10^-7^) from the SNP by asthma interaction analysis in MESA**

| SNP | Chr. | Position^a^ | Gene | Distance to gene | min./maj. | MAF | βinteraction (95%CI) | p |
| --- | --- | --- | --- | --- | --- | --- | --- | --- |
| rs41361551 | 3 | 7671257 | *GRM7* | 0 | T/C | 0.04 | 6.31 (3.95, 8.67) | 1.72E-07 |
| rs3804843 | 3 | 7673710 | *GRM7* | 0 | T/G | 0.04 | 6.73 (4.37, 9.09) | 2.62E-08^b^ |
| rs13099786 | 3 | 64770832 | *ADAMTS9-AS2* | 0 | A/T | 0.12 | 3.71 (2.31, 5.11) | 2.33E-07 |
| rs10250689 | 7 | 10120482 | *HSPA8P8* | -330754 | C/T | 0.09 | 4.66 (2.98, 6.34) | 5.91E-08^b^ |
| rs10254939 | 7 | 10121731 | *HSPA8P8* | -329505 | G/A | 0.09 | 4.58 (2.91, 6.26) | 8.94E-08 |
| rs17162655 | 7 | 10121991 | *HSPA8P8* | -329245 | T/A | 0.07 | 4.66 (2.85, 6.47) | 4.71E-07 |
| rs2041510 | 7 | 10128662 | *HSPA8P8* | -328726 | G/A | 0.07 | 4.91 (3.08, 6.73) | 1.50E-07 |
| rs4725259 | 7 | 10137696 | *HSPA8P8* | -313540 | T/C | 0.13 | 3.80 (2.42, 5.19) | 8.21E-08 |
| rs9640138 | 7 | 10145020 | *HSPA8P8* | -306216 | G/A | 0.12 | 3.80 (2.41, 5.20) | 1.08E-07 |
| rs4412254 | 7 | 67861492 | *LOC102723427* | -162999 | C/T | 0.06 | 5.04 (3.22, 6.86) | 5.99E-08^b^ |
| rs2865108 | 7 | 67861752 | *LOC102723427* | -162739 | C/A | 0.07 | 4.71 (2.93, 6.50) | 2.49E-07 |
| rs7798384 | 7 | 67864534 | *LOC102723427* | -159957 | C/T | 0.07 | 4.72 (2.93, 6.50) | 2.38E-07 |
| rs12667421 | 7 | 67893202 | *LOC102723427* | -131289 | T/C | 0.05 | 5.04 (3.11, 6.98) | 3.54E-07 |
| rs2865109^c^ | 7 |  |  |  | T/C | 0.06 | 5.04 (3.22, 6.86) | 5.99E-08^b^ |
| rs605571 | 9 | 4124542 | *GLIS3* | 0 | A/G | 0.41 | -2.42 (-3.33, -1.51) | 2.34E-07 |
| rs7067647 | 10 | 115698215 | *ATRNL1* | 0 | G/A | 0.38 | -2.58 (-3.55, -1.61) | 2.11E-07 |
| rs7914798 | 10 | 115706075 | *ATRNL1* | 0 | T/G | 0.38 | -2.57 (-3.54, -1.60) | 2.28E-07 |
| rs7904383 | 10 | 115716396 | *ATRNL1* | 0 | G/A | 0.38 | -2.66 (-3.63, -1.70) | 7.42E-08 |
| rs10510011 | 10 | 115719040 | *ATRNL1* | 0 | G/A | 0.38 | -2.66 (-3.62, -1.69) | 8.22E-08 |
| rs2286631 | 10 | 115727400 | *ATRNL1* | 0 | G/T | 0.40 | -2.63 (-3.59, -1.66) | 9.56E-08 |
| rs10134270 | 14 | 97305097 | *LOC101929241* | -153719 | A/C | 0.05 | 5.78 (3.55, 8.01) | 3.97E-07 |
| rs11632275 | 15 | 37285693 | *MEIS2* | 184394 | C/T | 0.39 | 2.50 (1.54, 3.46) | 3.50E-07 |
| rs2107212 | 17 | 40949109 | *KRT39* | -9308 | A/G | 0.20 | 2.96 (1.82, 4.09) | 3.72E-07 |
| rs2158297 | 17 | 40949294 | *KRT39* | -9123 | A/C | 0.20 | 3.08 (1.91, 4.25) | 2.64E-07 |
| rs12601191 | 17 | 40951857 | *KRT39* | -6560 | C/A | 0.20 | 2.98 (1.84, 4.12) | 2.99E-07 |
| rs10491138 | 17 | 40954668 | *KRT39* | -3749 | A/G | 0.18 | 3.29 (2.05, 4.53) | 2.02E-07 |
| rs17843024 | 17 | 40959087 | *KRT39* | 0 | A/C | 0.18 | 3.28 (2.04, 4.52) | 2.21E-07 |
| rs16968877 | 17 | 40959631 | *KRT39* | 0 | A/G | 0.17 | 3.31 (2.07, 4.55) | 1.76E-07 |
| rs10491137 | 17 | 40966545 | *KRT39* | 0 | A/G | 0.18 | 3.30 (2.06, 4.54) | 1.84E-07 |
| rs17411883 | 20 | 57552252 | *LOC101927842* | -2806 | C/T | 0.11 | 4.03 (2.57, 5.49) | 6.72E-08^b^ |
| rs17411904 | 20 | 57552874 | *LOC101927842* | -2184 | C/T | 0.11 | 4.04 (2.58, 5.50) | 6.09E-08^b^ |

A genome-wide SNP by asthma interaction analysis using linear regression modeling was performed for BMI as the outcome, among MESA subjects. Models included main effects for SNP and asthma, a SNP x asthma interaction term, and covariates to adjust for age, sex, and the first principal component. SNPs were coded additively as 0, 1, 2, according to the number of minor allele copies. Genome-wide suggestive SNP-asthma interactions in MESA are ordered by SNP chromosome numbers and positions. Parameter inferences are shown for SNP main effects and SNP-asthma interaction terms.

^a^ SNP positions are based on the GRCh38 assembly.

^b^ Genome-wide significant SNP-asthma interactions (p<6.93×10^-8^).

^c^ Not mapped to any assembly.

Abbreviations are as follows: chr, chromosome; dist. to gene, distance to gene; min./maj., minor/major allele; MAF, minor allele frequency.

**Table D. Replication results of genome-wide suggestive interacting SNPs at 17q21.2 in FHS and their meta-analysis results from MESA and FHS**

|  |  | FHS |  |  | Meta-analysis (fixed) | | Meta-analysis (random) | | | Heterogeneity | | |
| --- | --- | --- | --- | --- | --- | --- | --- | --- | --- | --- | --- | --- |
| SNP | Distance to *KRT39* | β (95%CI) | p-value | β (95%CI) | | p-value | | β (95%CI) | p-value | | I^2^ | p-value |
| rs2107212 | *9kb downstream* | 1.49 (0.37, 2.61 ) | 9.38E-03 | 2.21 (1.41, 3.01) | | 5.60E-08 | | 2.22 (0.78, 3.66) | 2.51E-03 | | 69.23% | 0.07 |
| rs12601191 | *7kb downstream* | 1.47 (0.35, 2.59) | 1.04E-02 | 2.21 (1.42, 3.01) | | 5.48E-08 | | 2.22 (0.74, 3.70) | 3.35E-03 | | 71.07% | 0.06 |
| rs2158297 | *9kb downstream* | 1.40 (0.30, 2.50) | 1.29E-02 | 2.19 (1.39, 2.99) | | 8.66E-08 | | 2.23 (0.58, 3.87) | 7.96E-03 | | 76.17% | 0.04 |
| rs17843024 | *Intron* | 1.38 (0.22, 2.54) | 2.00E-02 | 2.27 (1.42, 3.12) | | 1.49E-07 | | 2.32 (0.45, 4.18) | 1.47E-02 | | 79.25% | 0.03 |
| rs10491138 | *4kb downstream* | 1.36 (0.20, 2.52) | 2.21E-02 | 2.26 (1.41, 3.11) | | 1.62E-07 | | 2.31 (0.41, 4.20) | 1.67E-02 | | 79.91% | 0.03 |
| rs10491137 | *Intron* | 1.35 (0.18, 2.50) | 2.33E-02 | 2.26 (1.42, 3.11) | | 1.65E-07 | | 2.31 (0.40, 4.22) | 1.81E-02 | | 80.42% | 0.02 |
| rs16968877 | *Intron* | 1.21 (0.02, 2.40) | 4.67E-02 | 2.22 (1.36, 3.08) | | 4.07E-07 | | 2.25 (0.19, 4.31) | 3.20E-02 | | 82.60% | 0.02 |

Interaction term estimates (beta values and 95% CI) of replicable SNP-asthma interactions in region 17q21.2 from FHS. MESA and FHS were combined by meta-analysis, using both random effect and fixed effect models. SNPs are ordered according to their random effect model p-values from the most to the least significant.

**Table E. BMI value of rs2107212 genotypes by asthma status in MESA and FHS**

| Study |  | Genotype | | | p-value | | |
| --- | --- | --- | --- | --- | --- | --- | --- |
|  |  | AA (mean±SEM) | AG (mean±SEM) | GG (mean±SEM) | AA vs.GG | AG vs.GG | AA vs.AG |
| MESA | non-asthma | 26.98±0.83 | 27.25±0.38 | 27.80±0.25 | 0.067 | 0.019 | 0.565 |
|  | asthma | 33.99±3.86 | 30.24±1.25 | 28.06±0.90 | 0.014 | 6.33×10^-3^ | 0.095 |
|  | p-value | 5.23×10^-3^ | 1.99×10^-5^ | 0.571 |  |  |  |
| FHS | non-asthma | 27.64±1.84 | 27.64±0.51 | 27.81±0.38 | 0.858 | 0.602 | 0.997 |
|  | asthma | 32.32±4.10 | 29.85±1.07 | 28.80±0.69 | 0.126 | 0.107 | 0.277 |
|  | p-value | 0.060 | 3.54×10^-4^ | 0.013 |  |  |  |

**Table F. The association of each additional minor allele in rs2107212 and obesity with different BMI cut-offs**

A: in the asthmatic group

| study | BMI cut-off for obesity | | | | | |
| --- | --- | --- | --- | --- | --- | --- |
|  | >=30 kg/m^2^ | | >=28 kg/m^2^ | | >=25 kg/m^2^ | |
|  | OR | 95%CI | OR | 95%CI | OR | 95%CI |
| MESA | 2.30 | (1.43, 3.69) | 2.43 | (1.49, 3.96) | 3.74 | (1.89, 7.42) |
| FHS | 1.65 | (1.11, 2.46) | 1.50 | (1.00, 2.24) | 1.89 | (1.10, 3.27) |
| Meta-analysis | 1.89 | (1.39, 2.57) | 1.82 | (1.34, 2.49) | 2.47 | (1.61, 3.79) |

B: in the non-asthmatic group

| study |  | | BMI cut-off for obesity | |  | |
| --- | --- | --- | --- | --- | --- | --- |
|  | >=30 kg/m^2^ | | >=28 kg/m^2^ | | >=25 kg/m^2^ | |
|  | OR | 95%CI | OR | 95%CI | OR | 95%CI |
| MESA | 0.87 | (0.73, 1.04) | 0.82 | (0.70, 0.96) | 0.88 | (0.75, 1.04) |
| FHS | 0.94 | (0.73, 1.21) | 1.05 | (0.83, 1.32) | 0.91 | (0.71, 1.17) |
| Meta-analysis | 0.89 | (0.77, 1.03) | 0.88 | (0.78, 1.01) | 0.89 | (0.78, 1.02) |

The odds ratio of being obese for each additional minor allele (A allele) in rs2107212, stratified by asthma status in MESA, FHS, and meta-analysis (fixed effect model).

**Table G. BMI value of rs2107212 genotypes by asthma status in 1979 and 2005 in FHS**

| Year | non-asthma | | asthma | |
| --- | --- | --- | --- | --- |
|  | AA/AG (mean±SEM) | GG (mean±SEM) | AA/AG (mean±SEM) | GG (mean±SEM) |
| 1979 | 25.32±0.47 | 25.06±0.33 | 26.48±1.39 | 26.15±0.98 |
| 2005 | 27.64±0.52 | 27.77±0.41 | 30.27±1.85 | 29.39±1.31 |

**Table H. Net BMI change from 1979 to 2005 for rs2107212 genotypes by asthma status in FHS**

|  | AA/AG (mean±SEM) | GG  (mean±SEM) | p-value |
| --- | --- | --- | --- |
| non-asthma | 2.31±0.37 | 2.71±0.29 | 0.099 |
| asthma | 3.79±1.25 | 3.24±0.95 | 0.495 |
| p-value | 0.031 | 0.300 |  |

**Table I. Power analysis for MESA**

|  | MAF=0.05 | MAF=0.10 | MAF=0.15 | MAF=0.20 | MAF=0.25 | MAF=0.30 |
| --- | --- | --- | --- | --- | --- | --- |
| β_interaction_=2 | 0.0002 | 0.0020 | 0.0084 | 0.0215 | 0.0412 | 0.0649 |
| β_interaction_=3 | 0.0040 | 0.0536 | 0.1911 | 0.3784 | 0.5531 | 0.6850 |
| β_interaction_=4 | 0.0411 | 0.3692 | 0.7468 | **0.9244** | **0.9797** | **0.9944** |
| β_interaction_=5 | 0.2072 | **0.8303** | **0.9870** | **0.9994** | **0.9999** | **0.9999** |

Power was estimated using Quanto (available at http://biostats.usc.edu/software). Type I error (α) was set as 0.05/721,893= 6.93×10^−8^. Power estimates >= 0.80 are in bold. Parameters: sample size: 2474 individuals; Hypothesis: Gene-environment interaction; asthma prevalence: 0.1; BMI mean: 27.74; BMI standard deviation: 5.08; main effect for each gene (β): 0.5; main effect for asthma (β): 1.5.
